# Effects of music therapy as an alternative treatment on depression in children and adolescents with ADHD by activating serotonin and improving stress coping ability

**DOI:** 10.1186/s12906-022-03832-6

**Published:** 2023-03-06

**Authors:** Jong-In Park, In-Ho Lee, Seung-Jea Lee, Ryeo-Won Kwon, Eon-Ah Choo, Hyun-Woo Nam, Jeong-Beom Lee

**Affiliations:** 1 Department of Physiology, College of Medicine, Soonchunhyang University, Cheonan, 31151 Republic of Korea; 2 Department of Medical Sciences, Graduate School, Soonchunhyang University, Asan, 31538 Republic of Korea; 3 Department of Occupational and Environmental Medicine, Soonchunhyang University Cheonan Hospital, Cheonan, 31151 Republic of Korea

**Keywords:** Children and adolescents with ADHD, Music therapy, Serotonin (5-HT), Cortisol, BP, HR, Depression, Ability to address stress

## Abstract

**Objective:**

The objective of this study was to determine the effect of music therapy as an alternative treatment on depression in children and adolescents with attention-deficit hyperactivity disorder (ADHD) by activating serotonin (5-HT) and improving stress coping ability.

**Methods:**

This study is designed based on randomization method. A total of 36 subjects participated in the experiment, consisting of an ADHD control group (*n* = 18) and ADHD music therapy group (*n* = 18). The ADHD control group received standard care, while the ADHD music therapy group received music therapy and standard care. The ADHD music therapy group received both active music therapy (improvisation) and receptive music therapy (music listening) for 50 minutes, twice a week, for 3 months: a total of 24 times. From a neurophysiological perspective, changes in depression and stress were tracked by measuring 5-HT secretion, cortisol expression, blood pressure (BP), heart rate (HR), and CDI and DHQ psychological scales.

**Results:**

The ADHD music therapy group’s 5-HT secretion increased (*p* < 0.001), whereas cortisol expression (*p* < 0.001), BP (*p* < 0.001) and HR (*p* < 0.001) decreased. The CDI and DHQ psychological scales also showed positive changes (*p *< 0.01 and *p *< 0.001, respectively). However, the ADHD Con G’s (who did not receive music therapy) 5-HT secretion did not increase, whereas cortisol expression, BP, and HR did not decrease. In addition, the CDI and DHQ psychological scales did not display positive changes.

**Conclusions:**

In conclusion, the application of music therapy as an alternative treatment for ADHD children and adolescents showed positive neurophysiological and psychological effects. Therefore, this study would like to propose a new alternative to medicine for preventing and treating depression through various uses of music therapy.

## Introduction

Attention-deficit hyperactivity disorder (ADHD) is characterized by the presence of attention deficit, hyperactivity, and impulsivity as major symptoms, along with characteristics overlapping with other psychiatric disorders such as anxiety, depression, defiant disorder, and learning disability [1–3]. Compared with adolescents without ADHD, adolescents with ADHD show functional impairments across several areas (e.g., social, family, and academic fields) and experience reduced quality of life [4]. This, as a result, suggests that most adolescents and children with ADHD are more likely to develop depression [5–7].

From a physiological point of view, ADHD patients with depression show lower blood levels of serotonin (5-HT) than those without depression, suggesting that chronic reduction in the availability of 5-HT is associated with clinical manifestations of ADHD [8]. It is known that 5-HT can interact with the dopamine system to mediate the impulsive behavior. It is related to various behaviors such as impulsivity, inhibition, and attention [9]. In addition, 5-HT activity in the frontal-frontal circuitry is associated with the pathophysiological mechanism of ADHD. It is closely related to the secretion of 5-HT from serotonin neurons located in the central nervous system [10–12]. Depletion of 5-HT can lead to a lack of emotional control, resulting in depression [13]. Therefore, selective serotonin reuptake inhibitor (SSRI), a drug that regulates 5-HT, is considered a treatment of choice for ADHD treatment.

The environmental factors of depression in children and adolescents are mainly related to stress levels, and hormone cortisol is involved in controlling the level of stress [14]. Acute stress can trigger the release of adrenocorticotropin from the pituitary gland, which can stimulate the adrenal gland and release glucocorticoid (mainly human cortisol) into the bloodstream [15]. Therefore, high stress level led to high level of cortisol and at the same time causes various problems such as growth and immunity problems [16, 17].

Overall, children and adolescents with ADHD have high levels of stress but less 5-HT secretion than ordinary children [8] They are more likely to suffer from depression when they grow into adults [6, 18–20]. ADHD with depression as a coexisting disorder can be seen as a more serious situation than general ADHD [21, 22]. This is because accompanying depression can cause negative effects and risks on the patient’s social performance such as family problems, reduced opportunities for occupational activities, and increased likelihood of crime [23, 24].

Children and adolescents have much greater emotional and physical flexibility than adults. Thus, if appropriate treatment is performed children and adolescents with ADHD before they develop adult depression, the therapeutic effect can be much higher.

However, in reality, medical treatment for depression in ADHD mostly uses drugs. This is a symptomatic treatment that only temporarily suppresses symptoms. It has many limitations and problems because of the risk of side effects due to chemical drug treatment. There is an absolute need for a sustainable treatment that is safer with a lasting effect.

Music therapy is one of various nonpharmacological treatments. Numerous prior studies have already reported that music therapy performed by credentialed therapists can improve cognitive and sensory movements of depressed patients an restore self-esteem by establishing positive self-awareness [25, 26].

The purpose and approach of this study are designed in this context. The basic principle is described as follows. Children and adolescents with ADHD respond more sensitively to the unpredictability of daily life and experience more tension and stress than ordinary children and adolescents. When music therapy is applied, the subject of treatment projects his or her tension and anxiety into the musical structure. Through the process of developing harmonious music balance and chord, introjection of stable emotions is made [27–29]. As the emotional response gradually shifts from a tense fast tempo and dissonance to a slow tempo and consonance, unpleasant emotions are converted into pleasant emotions following the tempo and chord of the music [30, 31].

In addition, music therapy needs subjects to directly participate, such as improvisation and positive self-expression, which can not only further enhance the therapeutic effect, but also can alleviate depression by improving their ability to cope with stress. In particular, during the selection process of music used in music therapy, auditory stimulus factors such as tempo, chord, tone, and contrast effect of emotion should be considered.

The principle of operation is explained in detail as follows. Music therapy can produce substantial physiological changes. It can activate the physical therapeutic effect by generating a limbic response using musical sound [32]. The brain’s limbic system is closely related to respiratory rate, blood pressure, and heart rate. Applications of the auditory stimuli can relieve psychological stress experiences and depression [33].

Above all, negative emotions, and experiences such as depression as well as the ability to cope with the resulting stress should be considered. It is also a very important treatment goal in terms of social protection of children and adolescents with ADHD. Coping Skill refers to the ability to balance the secretion of hormones and neurotransmitters with the operation of the body immune system [34–36]. This adjustment process is also specifically related to 5-HT [37]. Related previous studies have confirmed that auditory stimulation using melody can directly affect monoamine activity in nucleus acumens. Furthermore, it has been found that auditory stimulation can activate the secretion of serotonin [38, 39]. Yet to date, ADHD is a neurodevelopmental disorder with an estimated worldwide prevalence of 3 to 7% in childhood and adolescence [40–42], and 3% in adulthood [43, 44], Prior studies lacked systematic investigation on the practice of music therapy for preventing depression as a major coexisting disease in children and adolescents with ADHD.

**Scheme 1:**
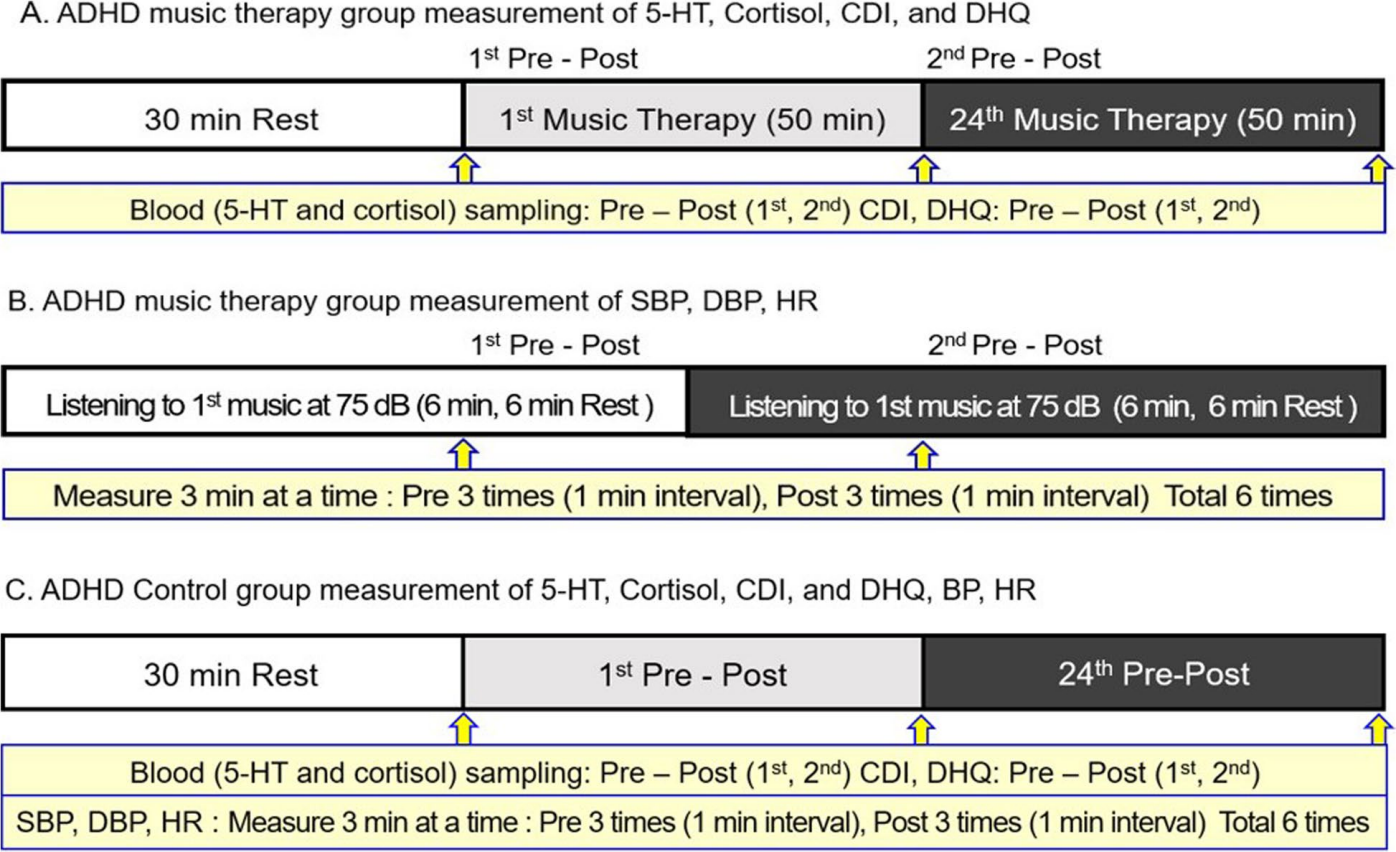
**A**: ADHD music therapy group: Before application of music therapy, in a condition stabilization situation after 30 minutes of rest, 5-HT was analyzed by collecting blood twice for each session (1st) and 24th session before execution and immediately after music therapy (2nd). Cortisol was analyzed through saliva collection under the same conditions. Psychological measurements (CDI, DHQ) were performed 24 times after the end of the program before music therapy was applied. **B**: ADHD music therapy group: SBP and DBP were measured before and after music therapy application. HR measurement was performed by naturally sitting on a chair with knees folded after listening to music for 2 minutes. Measurements were performed twice before and after music therapy. The average value of measurement results was recorded as a numerical value and analyzed. **C**: ADHD control group: Measurements carried out using the same method as the music therapy group but without the application of music therapy. 5-HT, serotonin; SBP: systolic blood pressure; DBP: diastolic blood pressure; HR: heart rate; CDI: Children’s Depression Inventory; DHQ: Daily Hassles Questionnaire

**Scheme 2:**
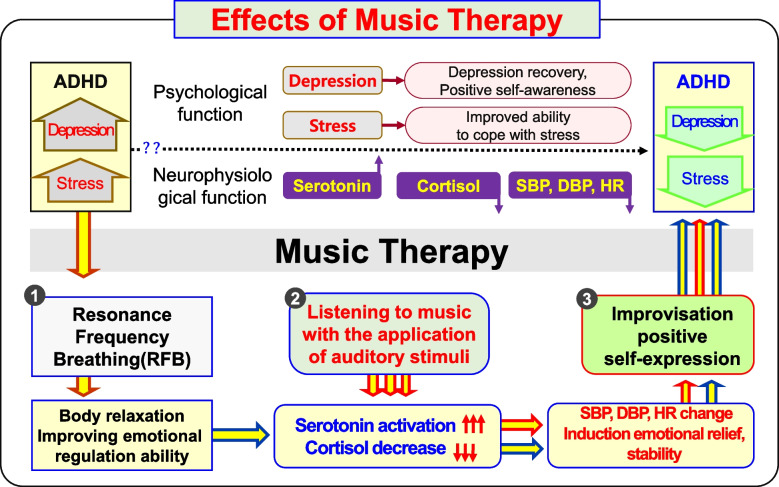
Alternative to Depressive Stress-Application Procedures and Effects of Music Therapy (Dotted line?? Reference). Step 1: Resonance frequency breathing [45, 46] (8 min). While listening to music with a stable tempo (60–80 bpm), resonance frequency breathing is performed, inducing stability relaxation and emotion control. Step 2: Listening to music with the applications of auditory stimuli receptive music therapy (music listening) (12 min). Based on emotional stability effect aroused by internal projection of depression into the musical structure, elements of auditory stimuli (tempo, tonality, chord, contrast effect of emotions) [30, 31] are applied, inducing 5-HT activation and cortisol decrease. Step 3: Improvisation and positive self-expression activities based on music as an auditory stimulus [47] active music therapy (improvisation) (30 min). This step aims to induce decreases of SBP, DBP and HR by improving positive self-awareness and stress coping skills. After the three-step music therapy is applied, psychological effects can be induced based on neurophysiological effects. 5-HT, serotonin; SBP: systolic blood pressure; DBP: diastolic blood pressure; HR: heart rate

Thus, the study attempts to quantitatively analyze effects of music therapy as an alternative treatment on depression in children and adolescents with ADHD based on changes in neurophysiological indicators of 5-HT and cortisol, systolic blood pressure (SBP), diastolic blood pressure (DBP), heart rate (HR), and psychological scale as psychological indicators (Scheme 1 and Scheme 2). Based on verified research results, we intend to confirm the possibility of continuous application and utilization of music therapy as an alternative to medicine for preventing and treating depression in children and adolescents with ADHD.

## Methods

### Trial design

The study was conducted near Chungcheong-do, Republic of Korea. Participants were recruited by posting a recruitment notice at the General Welfare Center located in Republic of Korea. This study is designed based on randomization method.

### Participants

The study was conducted near Chungcheong-do, Republic of Korea. Participants were recruited by posting a recruitment notice at the General welfare center located Chungcheong-do in Republic of Korea. There were four prerequisites for being selected as a subject for the experiment: (1) Children and adolescents must meet ADHD diagnostic criteria [2]. (2) Patients aged 7 to 8 must be diagnosed with ADHD (at a university hospital located near Chungcheong-do, Republic of Korea) and complaining of depression (with CDI Scale results at 22 pts. or higher) (3) ADHD children and adolescents must have obtained parents’ consent to participate in this study (4) ADHD children and adolescents must not have had any prior experience with music therapy.

Exclusion criteria are as follows: (1) ADHD children and adolescents who have experienced music therapy (2) ADHD children and adolescents with physical disabilities (3) Children and adolescents with ADHD with communication and self-report disorder. (4) Children and adolescents in the ADHD music therapy group who have participated in music therapy programs fewer than 3 times (5) ADHD children and adolescents who have experienced side effects and adverse reactions during study.

### Sample size

**Fig. 1:**
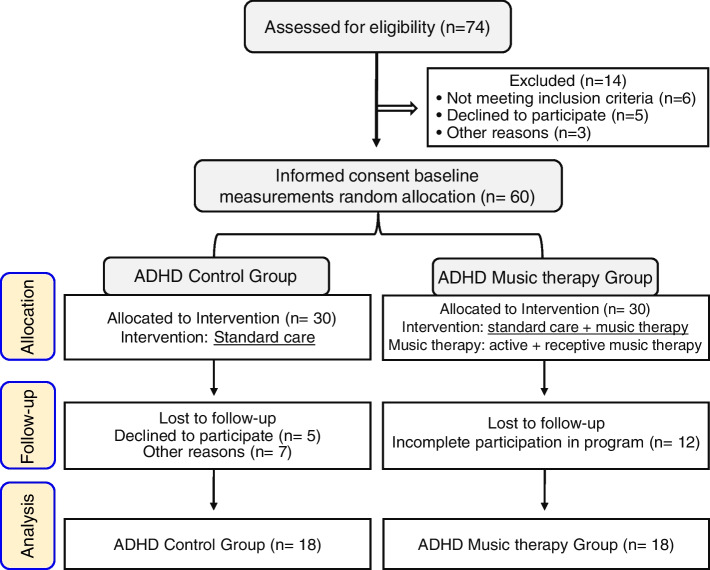
Flow chart: Participant flow

A total of 60 subjects were included in the design criteria for randomization in this study. They were divided into an ADHD control group (*n* = 30) and an ADHD music therapy group (n = 30) each on a 1:1 basis. (Fig. 1). By substituting a 0.05 confidence interval, a study power of 0.80, and effect size of 0.89 based on a previous study [48] using the G-power 3.1.9.7 program, a sample size of 42 people (ADHD control group 21/ADHD music therapy group *n* = 21) was calculated (Fig. 1). However, 18 ADHD control group subjects and 18 ADHD music therapy group subjects were finally selected due to the limitations of continuous therapy, COVID-19, and distance (Fig. 1). The ADHD control group received standard care, while ADHD the music therapy group received standard care and active music therapy (improvisation) and receptive music therapy (music listening) and completed statistical analysis after follow-up (2 months). The study flowchart is summarized in (Fig. 1).

### Randomization and blinding

The principal Investigator selected the subjects by reviewing the selection and exclusion criteria among ADHD children and youth participants. Random assignments were made using random number table generated by the Excel program to generate a random list with 2 groups and 60 samples.

The registered subjects were equally assigned to the ADHD Control group or ADHD music therapy group assigned by the random number table in the order of registration. This study is a music therapy intervention study. Therefore, the principal investigator of the ADHD children and youth participants, music therapists, and research will not be blinded by the assigned treatment. However, data statisticians will be blinded to group allocation. In order to protect the study subjects as much as possible, alphabet letters replaced their names.

#### Interventions

ADHD control group: standard care, ADHD music therapy group: standard care and active music therapy (improvisation) and receptive music therapy (music listening).

### Music therapy intervention

Music therapy, an alternative to medication, was conducted for 3 months (24 sessions in total) twice a week (Tuesday and Friday) from 2:00 PM to 3:00 PM each time. During this period, music therapy was conducted for 50 minutes for the ADHD music therapy group. Music therapy was conducted as follows step1: 8 min, step2: 12 min, and step3: 30 min (total 50 min). The music therapy program referred to [30, 31, 45–47, 49–51] previous studies to restore psychological and neurobiological functions to relieve depression and stress according to the purpose of this study. In addition, the researcher reconstructed it appropriately for the subject, including both receptive (listening to music) and active music therapy (Improvisation) interventions, and it was conducted in three stages after verifying the validity of three music therapy experts (Scheme 1). Application of music therapy was conducted by three certified music therapists who had extensive clinical therapy experience.

Music therapy was conducted in a stable and comfortable treatment room without noise, maintaining room temperature of 20 ~ 22 °C to get the most precise measurements. In the music therapy room, a piano for improvisation and a variety of rhythmic instruments that are easy to use to improvise without any special skills were placed.

When listening to auditory stimulus music, the subjects closed the door and sat in a comfortable position on the chair. And they closed eyes and listened to music, recalling positive images and words according to the therapist’s guidance. 3 steps of activity were conducted by using positive image and word that are visualized during listening to auditory stimulus music. 3 music therapists guided subjects to visualize positive images and words while they were listening to music.

Also, the therapists recorded specific changes in the subject’s behavior and attitude. After finishing each session of therapy, they qualitatively classified emotions expressed based on positive self-expression.

After music therapy, a song file for music appreciation was transmitted to ADHD Music therapy Group so that the song could be enjoyed at home. During the 3 months / 24 sessions of music therapy, music appreciation was practiced in daily life for 5 days a week when treatment was not performed, and it was intended to increase the effectiveness of the treatment in connection with the home.

### Music preference survey

Specifically, this study attempted to select songs that correspond to three music conditions (Motivating, Relaxing, and M + R) among music that is highly preferred by Korean children and adolescents. Along with the selection of healing music, the overall structural analysis of the song was evaluated by a group consisting of two composers and one professional performer.

The subjects who finally decided to participate in the ADHD music therapy group (*n* = 18) completed a preference questionnaire consisting of a 5-point Likert Scale, and audio files were sent using a smartphone or email. To minimize the influence of lyrics, genres like pop without lyrics, traditional Korean music, and New Age were selected.

Also, music that matched the participants’ preferences (having been checked as “normal” or “like” by them) was selected [52].

The music listening used for the music therapy consisted of Motivating, Relaxing, and M + R (a total of three songs were selected: Arirang, Golden Star, White Tower), and were selected through a preference survey. Based on a previous study [53], the chosen songs were composed in the key of C, D, and G Major, and mainly used major chords, with some minor chords interspersed to create interest in the music [53].

### Standard care

The ADHD control group children and adolescents continued to receive existing recommended [54], drug therapy only. ADHD music therapy group children and adolescents and continued to receive existing recommended [54], drug therapy and active music therapy (improvisation) and receptive music therapy (music listening).

## Evaluation

### Blood sampling

Blood samples were collected from antecubital veins of subjects according to the guidelines of the Clinical and Laboratory Standards Institute to identify changes in serum markers immediately before and after music therapy. Blood samples were transferred to SST tubes and immediately centrifuged at 3000 rpm for 10 minutes at 4 °C. The serum was subsequently removed and stored in 1 ml aliquots at − 80 °C until analysis (Scheme 2).

### Analysis of 5-HT and cortisol levels

To determine 5-HT level, a high-pressure liquid chromatography (HPLC) method (Serotonin Kit, Recipe, Germany) with an Alliauce Waters 465 HPLC (Waters, USA) was used. Cortisol level was determined using a CORTISOL RIA CT (AMP, Germany) with a γ-counter COBRA 5010 QUANTUM (PACKARD, USA).

### Measurement equipment for SBP, DBP and HR

SBP, DBP, and HR were measured using an automatic blood pressure monitor (OMRON, HEM-7210, Japan). SBP, DBP, and HR was measured one time per minute for a total three times immediately before and after music therapy. The average value was then calculated and used. All statistical analyses such as paired-test, unpaired-test, and analysis to verify music therapy follow-up effects were conducted using a commercial software (SPSS for Windows, version 21.0; SPSS Inc., Chicago, IL, USA). Results are expressed as average ± standard deviation. Significance level was set at *p* < 0.05 (Scheme 2).

### Psychological measurement usage inventory

#### Children’s depression inventory (CDI)

Children’s Depression Inventory employed Kovacs’s CDI; M Kovacs [55] infant depression inventory, which modified Back’s Depression inventory for use in age range of 7 to 17 years old. Depression inventory consisted of a total of 27 questions. Based on answers to each question, the status of depression was diagnosed by combining each score from 0 to 2. If the combined score was in the score range of 22 to 25, it indicated a somewhat depressed condition. If the combined score was in the score range of 26 to 28, it indicated a in quite depressed condition. If combined score was above 29, it indicated a harsh depressed condition [56].

#### Daily hassles questionnaire (DHQ)

Daily stress was assessed with a 16-item questionnaire. The Daily stress questionnaire was based on Daily Hassles Questionnaire by RT Rowlison and RD Felner [57], and revised by MH Han and AJ Yoo [58], for students in Republic of Korea.

Measurement of daily stress consists of eight questionnaires of parent’s factors, seven questionnaires of family environmental factors, seven questionnaires of friend’s factors, seven questionnaires related to study, and seven questionnaires related to teacher and school. Likert inventory has a full score of 4, with higher score indicating higher daily stress level perceived [59].

The internal consensus level (Cronbach’s) of each factor was 0.85 for parent-related stress, 0.83 for home environment-related stress, 0.89 for friend-related stress, 0.83 for academic-related stress, and 0.77 for stress related to teachers and school life.

### Ethics

This study has been approved by the Institutional Review Board on Human Subjects Research and Ethics Committees, Soonchunhyang University, Cheonan, Korea (Approval No. 1040875-202012-BR-095, 1040875-202009-BR-075 and 1040875-202104-BR-030). All subjects and their parent or legal guardian were thoroughly briefed on the purpose of the study, experimental procedures, and potential risks. Participants were children and adolescents between the ages of 9–15. Before the study was undergone, informed consent/assent to participate in the study was obtained from the participant’s parents or legal guardians of all the children and adolescents who took part in the study in the form of a statement in a manuscript. All procedures complied with the 2013 Helsinki Declaration of the World Medical Association.

### Statistical analysis

Technical statistics as a method of statistical analysis was conducted using paired-test, unpaired-test, and analysis to verify effects of music therapy during follow-up using a commercial computer software (SPSS for Windows, version 21.0; SPSS Inc., Chicago, IL, USA). Data are expressed was average ± standard deviation. Significance level was set at *p* < 0.05.

## Results

### Participant enrolment

With the participation of three music therapists, pre-interviews were conducted with 60 people who were recruited through the recruitment notice for music therapy research. Recruitment commenced in 2021 and was completed in 2022. A statistical analysis was performed at follow-up (2 months later). In this study, 60 participants were recruited and underwent both the target selection criteria and the exclusion criteria. Subsequently, subjects who did not participate in all sessions due to conflicting work schedules and experimental schedules were excluded from the study. Therefore, among the 60 subjects, a total of 36 subjects, 18 in the ADHD control group and 18 in the music therapy group, were classified as subjects who met the criteria for selection as subjects for research. The physical features of all subjects are shown in Table 1. This study clearly explained study purpose, process, method, and possible side effects in detail to parents, legal representatives, ADHD children, and adolescents. Also, parents’ and legal representative’s consent was obtained after they had been fully informed about the overall study process. During the study no abnormal reactions or side effects were found in any of the participants.

**Table 1:** Physical characteristics of subjects (*n* = 36)

Classification of participants	ADHD Control group		ADHD Music therapy group	
	Male = 10	Female = 8	Male = 13	Female = 5
Age	11.91 ± 2.77	12.43 ± 2.99	11.76 ± 2.34	12.53 ± 2.52
Height (cm)	144.45 ± 11.52	145.57 ± 11.06	143.87 ± 10.51	141.26 ± 9.44
Weight (kg)	42.53 ± 9.25	41.86 ± 9.51	41.82 ± 9.69	41.08 ± 8.35
BSA (m^2^)	1.30 ± 1.03	1.30 ± 0.01	1.29 ± 0.98	1.26 ± 0.97
BMI (kg/m^2^)	21.55 ± 2.58	21.29 ± 2.98	20.21 ± 2.33	20.59 ± 2.41
% fat	20.35 ± 3.11	20.43 ± 3.31	19.25 ± 3.04	19.98 ± 3.10

Values are presented as mean ± SD, standard deviation. *BSA* body surface area, *BMI* body mass index. BSA was calculated using the Du Bois formula [60]. Body fat percentage was measured using the bio-impedance method (InBody 520, Seoul, Korea)

#### Neurophysiological analysis

##### 5-HT

**Fig. 2:**
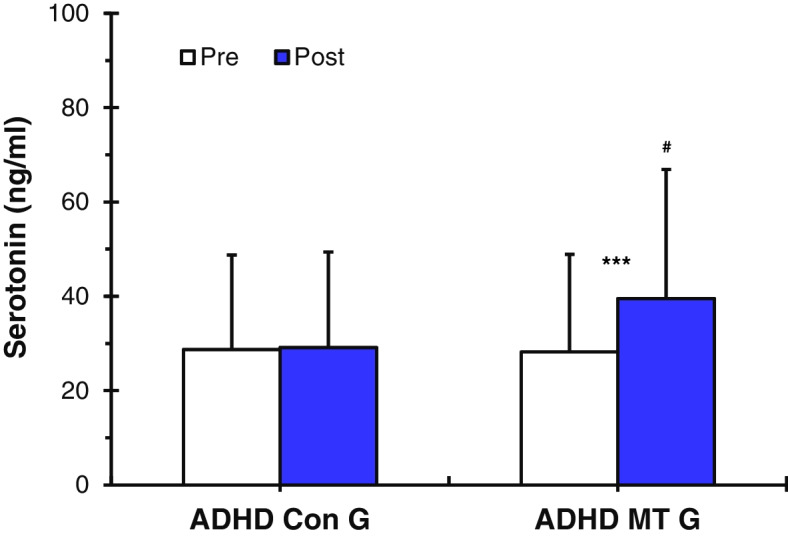
Values (*n* = 36) are presented as mean ± SD. Results of the serotonin (5-HT) analysis of the ADHD control group (ADHD Con G) and the ADHD music therapy group (ADHD MT G). The results of the analysis of the serotonin expression level of the ADHD MT G showed a statistically significant increase compared to before they had music therapy (***p < 0.001). However, the 5-HT analysis of the ADHD Con G showed that the serotonin expression levels had not significantly increased after, as compared to before. Statistically significant differences between ADHD Con G and ADHD MT G (#*p* < 0.05). Thus, the application of music therapy can contribute to 5-HT activation

The results of the analysis of the serotonin expression level of the ADHD music therapy group (ADHD MT G) that had music therapy showed a statistically significant increase after, as compared to before, they had music therapy (before: ADHD MT G: 28.20 ± 20.70 ng/ml vs. after: 39.50 ± 27.40 ng/ml *p* < 0.001, Fig. 2). However, the 5-HT analysis of the ADHD control group (ADHD Con G), who didn’t receive music therapy, showed that serotonin expression levels had not significantly increased after, as compared to before (before: 28.75 ± 20.03 ng/ml vs. after: 29.63 ± 20.29 ng/ml, Fig. 2). Prior to music therapy being applied, there was no significant difference in serotonin expression levels between the ADHD MT G and the ADHD Con G (before: ADHD MT G: 28.20 ± 20.70 ng/ml vs. ADHD Con G: 28.75 ± 20.03 ng/ml, Fig. 2). However, results of the analysis of the 5-HT expression levels after the ADHD MT G had music therapy showed that 5-HT expression was statistically significantly higher in the ADHD MT G than in the ADHD Con G (ADHD MT G: 39.50 ± 27.40 ng/ml vs. ADHD Con G: 29.63 ± 20.29 ng/ml, *p* < 0.05, Fig. 2).

##### Cortisol

**Fig. 3:**
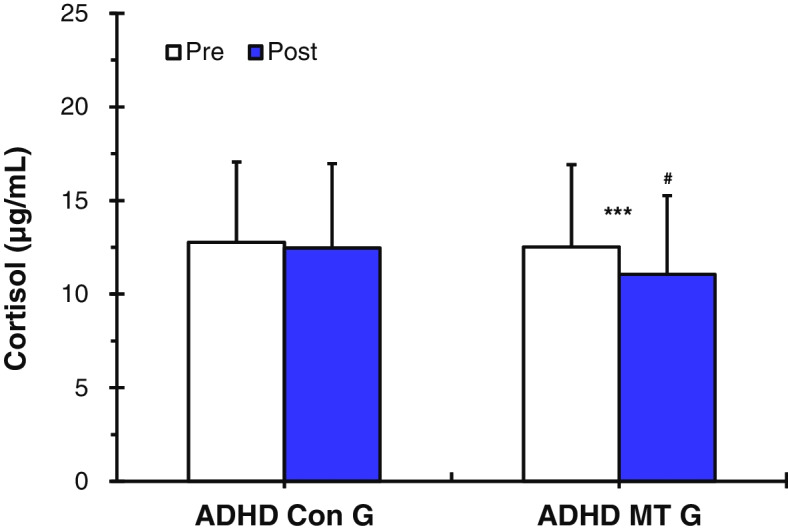
Values (*n* = 36) are presented as mean ± SD. Cortisol levels in the ADHD control group (ADHD Con G) and the ADHD music therapy group (ADHD MT G). The ADHD MT G showed statistically significantly lower cortisol levels after the music therapy (***p < 0.001). However, analysis of the ADHD Con G showed that cortisol level expression had not significantly decreased after, as compared to before. Statistically significant differences between ADHD Con G and ADHD MT G (#*p* < 0.05). Thus, the application of music therapy may contribute to a decrease in cortisol levels

The ADHD music therapy group (ADHD MT G) showed statistically significantly lower cortisol levels after the music therapy (before: 12.52 ± 4.40 ng/ml after g/dL vs. after: 11.06 ± 4.20 g/dL, *p* < 0.001, Fig. 3). However, the analysis of the ADHD control group (ADHD Con G), who did not receive music therapy, showed that cortisol level expression had not significantly decreased after, as compared to before (before:12.77 ± 4.29 g/dL vs. after: 12.46 ± 4.51 g/dL, Fig. 3). Prior to music therapy being given, there was no significant difference in cortisol expression levels between the ADHD MT G and the ADHD Con G (ADHD MT G: 12.52 ± 4.40 g/dL vs. ADHD Con G: 12.77 ± 4.29 g/dL, Fig. 3). However, results of the analysis of the cortisol expression levels of the ADHD MT G after receiving music therapy showed that cortisol expression was statistically significantly lower in the ADHD MT G than in the ADHD Con G (ADHD MT G: 11.06 ± 4.20 g/dL vs. ADHD Con G: 12.46 ± 4.51 g/dL, *p* < 0.05, Fig. 3).

##### SBP, DBP, and HR

The ADHD music therapy group (ADHD MT G) showed statistically significantly lower SBP, DBP, and HR levels after the music therapy (all *p* < 0.001, Table 2). However, analysis of the ADHD control group (ADHD Con G), who did not receive music therapy, showed that SBP, DBP, and HR levels were not significantly lower after, as compared to before (Table 2). Prior to music therapy being administered, there was no significant difference in SBP, DBP, and HR levels between the ADHD MT G and the ADHD Con G (Table 2). However, results of the analysis of the SBP, DBP, and HR levels after music therapy was given to the ADHD MT G showed that the SBP, DBP, and HR levels were all statistically significantly lower in the ADHD MT G than in the ADHD Con G (all *p* < 0.05, Table 2).

**Table 2:** Effect of music therapy on SBP, DBP and HR by ADHD control group and ADHD music therapy group (pre vs. post difference, paired t-test: pre vs. pre and post vs. post difference, unpaired t-test)

**Paired t-test**	**ADHD Con G**				**ADHD MT G**			
	**Pre**	**Post**	***t***	***p***	**Pre**	**Post**	***t***	***p***
SBP (mmHg)	127.29 ± 9.56	126.90 ± 9.07	0.88	0.915	127.05 ± 9.14	121.76 ± 9.34	6.02	0.000***
DBP (mmHg)	76.91 ± 9.11	76.71 ± 9.28	1.25	0.113	75.62 ± 8.02	72.43 ± 8.01	6.14	0.000***
HR (bpm)	93.43 ± 10.70	93.33 ± 10.80	1.00	0.615	93.71 ± 10.45	82.29 ± 9.76	5.45	0.000***
**Unpaired** ***t***-**test**	**ADHD Con G vs. ADHD MT G**				**ADHD Con G vs. ADHD MT G**			
	**Pre**	**Pre**	***t***	***p***	**Post**	**Post**	***t***	***p***
SBP (mmHg)	127.29 ± 9.56	127.05 ± 9.14	0.08	0.467	126.90 ± 9.07	121.76 ± 9.34	1.81	0.039*
DBP (mmHg)	76.91 ± 9.11	75.62 ± 8.02	0.49	0.314	76.71 ± 9.28	72.43 ± 8.01	1.69	0.048*
HR (bpm)	93.43 ± 10.70	93.71 ± 10.45	−0.09	0.465	93.33 ± 10.80	82.29 ± 9.76	3.48	0.001**

Values (*n* = 36) are presented as mean ± SD: standard deviation. *p < 0.05, **p < 0.01, and ***p < 0.001. Sub-categories; *SBP* systolic blood pressure, *DBP* diastolic blood pressure, *HR* heart rate. ADHD control group (ADHD Con G), ADHD music therapy group (ADHD MT G)

#### Psychological scale analysis

##### CDI

**Fig. 4:**
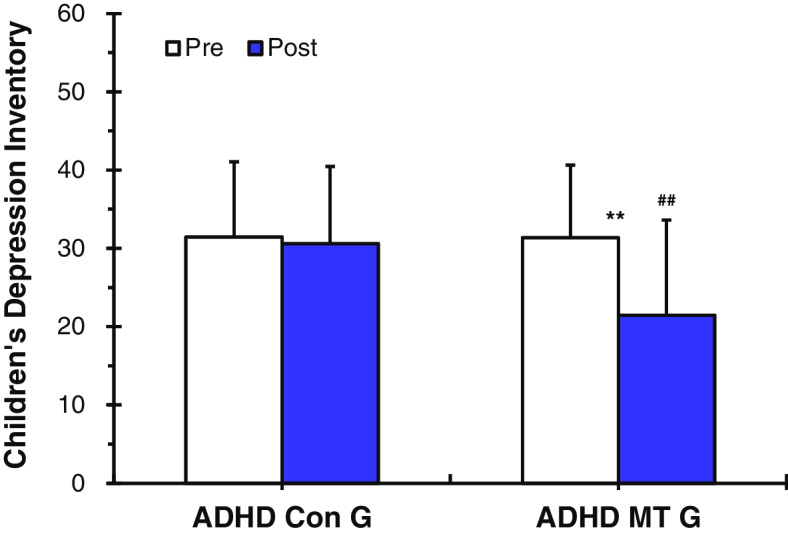
Values (*n* = 36) are presented as mean ± SD. Results of depression analysis according to Children’s Depression Inventory (CDI) scale: The ADHD control group (ADHD Con G) vs. the ADHD music therapy group (ADHD MT G). The ADHD MT G showed statistically significantly lower CDI depression scores after the music therapy (**p < 0.01). However, the ADHD Con G had CDI depression scores that were not significantly lower after, as compared to before. Statistically significant differences between ADHD Con G and ADHD MT G (##*p* < 0.01). The results confirm that the application of music therapy can contribute to the reduction of symptoms of depression in children and youths with ADHD

The ADHD music therapy group (ADHD MT G) showed statistically significantly lower CDI depression scores after the music therapy (*p* < 0.01, Table 3 and Fig. 4). However, the ADHD control group (ADHD Con G), who did not receive music therapy, had CDI depression scores that were not significantly lower after, as compared to before (Table 3 and Fig. 4). Prior to music therapy being applied, there was no significant difference in CDI depression scores between the ADHD MT G and the ADHD Con G (Table 3 and Fig. 4). However, results of the analysis of the CDI depression scores of the ADHD MT G after receiving music therapy showed that the CDI depression scores were statistically significantly lower in the ADHD MT G than in the ADHD Con G (*p* < 0.01, Table 3 and Fig. 4).

**Table 3:** Effect of music therapy on CDI and DHQ values by ADHD control group and ADHD music therapy group (pre vs. post difference, paired t-test: pre vs. pre and post vs. post difference, unpaired t-test)

**Paired** ***t*-test**	**ADHD Con G**				**ADHD MT G**			
	**pre**	**post**	***t***	***p***	**pre**	**post**	***t***	***p***
CDI scale	32.70 ± 10.21	32.40 ± 9.37	1.68	0.05	31.35 ± 9.27	21.45 ± 12.17	4.01	0.001**
DHQ scale	113.30 ± 26.15	110.95 ± 28.75	3.08	0.58	115.75 ± 30.02	80.50 ± 35.87	5.45	0.000***
**Unpaired** ***t*-test**	**ADHD Con G vs. ADHD MT G**				**ADHD Con G vs. ADHD MT G**			
	**pre**	**pre**	***t***	***p***	**post**	**post**	***t***	***p***
CDI scale	32.70 ± 10.21	31.35 ± 9.27	-0.31	0.45	32.40 ± 9.37	21.45 ± 12.17	2.53	0.008**
DHQ scale	113.05 ± 33.11	115.75 ± 30.02	0.28	0.39	110.95 ± 28.75	80.50 ± 35.87	3.07	0.002**

Values (*n* = 36) are presented as mean ± SD: standard deviation. **p < 0.01 and ***p < 0.001. Sub-categories; CDI: Children’s Depression Inventory, DHQ: Daily Hassles’ Questionnaire. ADHD control group (ADHD Con G), ADHD music therapy Group (ADHD MT G)

##### DHQ

**Fig. 5:**
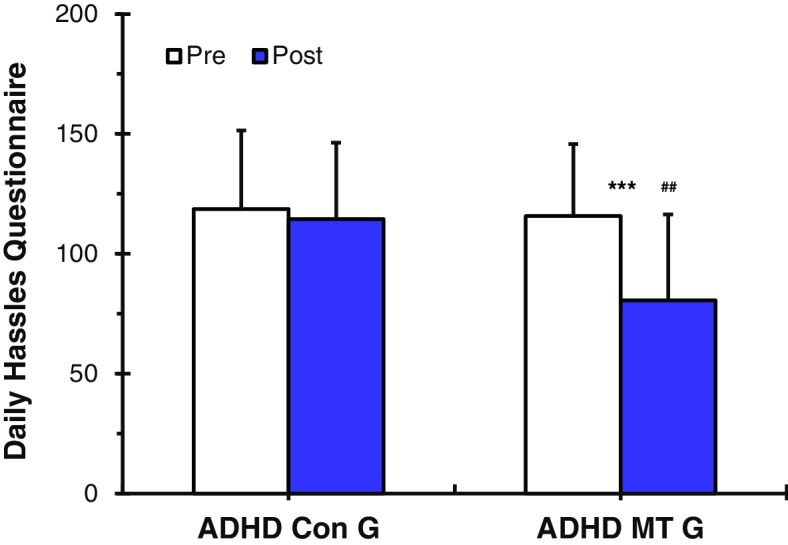
Values (*n* = 36) are presented as mean ± SD. Daily Hassles Questionnaire (DHQ) scale analysis: ADHD control group (ADHD Con G) vs. ADHD music therapy group (ADHD MT G). The ADHD MT G showed statistically significantly lower DHQ stress scale scores after the music therapy (***p < 0.001). However, the DHQ stress scale scores of the ADHD Con G were statistically significantly not lower after, as compared to before. Statistically significant differences between ADHD Con G and ADHD MT G (##*p* < 0.01). The results confirm that the application of music therapy can contribute to the reduction of symptoms of stress in children and youths with ADHD

The ADHD music therapy group (ADHD MT G) showed statistically significantly lower DHQ stress scale scores after the music therapy (*p* < 0.001, Table 3 and Fig. 5). However, the DHQ stress scale scores of the ADHD control group (ADHD Con G), who did not have music therapy, were statistically significantly not lower after, as compared to before (Table 3 and Fig. 5). Prior to music therapy being applied, there was no significant difference in the DHQ stress scale scores between the ADHD MT G and the ADHD Con G (Table 3 and Fig. 5). However, results of the analysis of the DHQ stress scale scores of the ADHD MT group after receiving music therapy showed that the DHQ stress scale scores were statistically significantly lower in the ADHD MT G than in the ADHD Con G (*p* < 0.01, Table 3 and Fig. 5).

## Discussion

This study was conducted to verify the effectiveness of music therapy as an alternative treatment for improving depression in children and adolescents with ADHD by serotonin activation and stress coping ability improvement. To this end, this study compared and analyzed neurophysiological and psychological parameters before and after music therapy for children and adolescents with ADHD by measuring 5-HT, cortisol, SBP, DBP, HR, and psychological scale (CDI, DHQ) (Scheme 2). For the music therapy treatment, resonance frequency breathing [45, 46], was applied as the first step. The second step involved using musical elements such as chords, tempo, tonality, and contrast effects of emotions [30] to enable the listening to have auditory stimulation elements. When applying the two-stage treatment, the goal was to activate depression-related 5-HT and decrease stress-related cortisol. The measurement results of 5-HT and cortisol in relation to the second step of the treatment are as follows: Prior to music therapy being applied, there was no significant difference in serotonin expression levels between the ADHD MT G and the ADHD Con G. However, results of the analysis of the 5-HT expression levels of the ADHD MT G after receiving music therapy showed that 5-HT expression was statistically significantly higher in the ADHD MT G than in the ADHD Con G (Fig. 2). The ADHD MT G showed statistically significantly lower cortisol levels after the music therapy: however, the cortisol levels of the ADHD Con G had not significantly decreased after, as compared to before (Fig. 3). The research plan was based on previous studies which showed correlations between musical stimuli and lowered depression and stress [46, 61]. Actual measurement results also showed positive changes in neurophysiology and psychology after treatment using music therapy. This is consistent with the results of a previous study conducted by [26, 28, 33, 61] suggesting that emotions and neurophysiological factors such as blood pressure, heart rate, hormones, and body temperature are closely related [62]. It can be seen that music therapy can directly cause neurophysiological changes in the human body and prove the possibility of switching in a positive direction. In addition, the ADHD MT G showed as a result of measurement, it was confirmed that 5-HT activation and cortisol reduction resulted in positive results in subsequent depression scale (CDI) test and related stress scale (DHQ) test (Table 3). It was confirmed that the neurophysiological effect of music therapy was interconnected with the psychological effect.

In the last step, after improvisation and positive self-expression activities were performed as post-activity, ADHD music therapy group showed statistically significantly lower SBP, DBP, and HR levels after the music therapy. In addition, as a result of measuring the psychological scale, it was confirmed that the score of the stress scale (DHQ) test of the ADHD MT G was decreased significantly after treatment than that before music therapy. Since those with ADHD are more likely to suffer from emotional disorders such as depression [63, 64], music therapy can be interpreted in a very encouraging direction in that it not only relieves ADHD children and adolescents’ depression, but also strengthens their ability to cope with problems. When overall analysis results are summarized, application points that can extend music therapy to the treatment process of depression and stress in ADHD children and youth groups can be summarized as follows. First, music therapy can be a non-pharmaceutical alternative treatment for depression and stress of ADHD. In fact, previous studies have consistently indicated that it is necessary to combine continuous and non-pharmaceutical treatments in order to reduce side effects of pharmacological treatment and increase the effectiveness of treatment for these diseases [2, 65]. Music therapy used in the present study has an advantage in that it has convenient accessibility during continuous treatment. Children and adolescents in the ADHD music therapy group In order to proceed with a music therapy, it was designed to transmit sound source files for listening to auditory music at home and allow them to observe their heart rates using home blood pressure and heart rate monitoring machines [66], which are recommended for ADHD children and adolescents after listening to music. This is an attempt to increase the treatment effect by linking music therapy to daily life. This music therapy configuration minimizes the need for space and cost for treatment. It can be applied more easily than other non-pharmacological treatments. Second, music therapy is an effective treatment method for developing the ability to cope with stress in children and adolescents with ADHD. In particular as social, and psychological stress increases due to the COVID-19 [67], treatment measures and their necessity are further emphasized [66, 68, 69]. The music therapy constructed and devised in this study is also very effective in this case. Music therapy has the advantage that it can be accessed from anywhere without any spatial constraints and that the treatment cost is low. In addition, music therapy can be conveniently used even under unexpected circumstances, such as a global pandemic. On the other hand, existing studies on music therapy for children and adolescents with ADHD have been focused on improving their academic ability [70, 71] or on the improvement of impulsivity and attention based on cognitive therapy [72–76]. On the contrary, this study has a major difference in that it focused on depression as the most fundamental emotional underlying disease and sought musical therapy as a solution to this.

In particular, the study quantified 5-HT and cortisol expression based on neurophysiological treatment effects, presented indicators on changes in SBP, DBP, and HR, and verified psychological treatment effects through psychological scale verification (CDI, DHQ). Therefore, it is expected that the applicable proposal of Scheme 2, an alternative model of alternative medicine, will be used in various ways for ADHD children and adolescents.

### Limitations

This study suggested the use of scientific clinical evidence in the form of physiological indicators as an alternative to depression and stress, which are emotional symptoms of ADHD. It is necessary to conduct a follow-up study on data analysis that can confirm the point of subject’s recovery when music therapy is applied to subjects suffering from depression and stress.

## Conclusion

In conclusion, the application of music therapy as an alternative treatment for depression in ADHD children and adolescents showed positive neurophysiological and psychological effects. This study intends to present a new alternative of medicine towards preventing and treating depression of ADHD through a neurophysiological and psychological approach. Indicators of the confirmed results meet the need for continuous application and the added value of use. Additionally, it is expected that music therapy will contribute to the establishment and spread of clinical foundations through active use in the medical field.

## Data Availability

All data generated or analyzed during this study are included in this published article. The raw data supporting the conclusion of this article will be made available by the authors, without undue reservation. The dataset supporting the conclusions of this article can be made available from the corresponding author on reasonable request.
